# The Association between Preoperative Serum C-Reactive Protein and Hepatocellular Carcinoma Recurrence in Patients with Chronic Hepatitis B Virus (HBV) Infection—A Retrospective Study

**DOI:** 10.1371/journal.pone.0116909

**Published:** 2015-01-20

**Authors:** Xiaoming Zhao, Jingyu Luo, Bobo Li, Shuguang Liu, Daotang Li

**Affiliations:** 1 School of Medicine and Life Sciences, University of Jinan and Shandong Academy of Medical Sciences, 106 Jiwei Road, Jinan City, Shandong Province, 250022, China; 2 Department of Thoracic Surgery, Shandong Cancer Hospital and Institute, 440 Jiyan Road, Jinan City, Shandong Province, 250117, China; Nanjing, CHINA

## Abstract

The prognosis of the patients with hepatocellular carcinoma (HCC) recurrence following curative hepatectomy is usually dismal. Whether preoperative serum C-reactive protein (CRP) can predict the recurrence of HCC in patients with chronic HBV infection is not clear. Total 232 patients with chronic HBV infection were included in this retrospective study. We investigated the association between detailed preoperative serum CRP levels and early (≤ 2 year) and late (> 2 year) HCC recurrence following curative hepatectomy. After adjusting for potential confounders, we found a saturation effect for preoperative serum CRP of 2.1 mg/dl existed for early HCC recurrence (ER). The incidence of ER increased with preoperative serum CRP less than 2.1 mg/dl (OR = 3.5, 95% CI 1.6–7.6, P = 0.001), and higher preoperative serum CRP (>2.1 mg/dl) did not increase the incidence of ER (OR = 0.8, 95% CI 0.2–2.7, P = 0.703). Whereas there is a linear relationship between preoperative serum CRP and late HCC recurrence (LR) (OR = 0.2, 95% CI, 0.1- 0.4) (OR = 1.8, 95% CI, 1.2–2.5, P = 0.002). In addition, the optimal cutoff point for serum CRP level was 1.5 mg/dl, instead of 1.0 mg/dl, in predicting both ER and LR. Patients with higher preoperative serum CRP level (>1.5 mg/dl) had lower recurrence free survival rates and overall survival rates (P<0.01). These results suggest that preoperative serum CRP played different roles on ER and LR following curative hepatectomy, thus further predictingthe prognosis in patients with chronic HBV infection.

## Introduction

Hepatocellular carcinoma (HCC) is the sixth most commonly diagnosed cancer and the third most common cause of cancer-related death worldwide[1]. Hepatectomy has been accepted as a curative treatment modality for HCC patients [2,3]. Despite advances in surgical techniques and perioperative treatment in the late period, tumor recurrence still remains high which hampers the long-term benefits of surgical treatment[4]. The cumulative 5-year recurrence rate is up to approximately 80%[4], while the overall survival is lower than 50% at 5 years[5].

C-reactive protein (CRP) is an acute-phase reactant that is synthesized by hepatocytes in response to the inflammatory reaction, and its production is regulated by proinflammatory cytokines [6]. In previous studies, serum CRP level has been found to be a prognostic indicator for gastric carcinoma [7], colorectal cancer [8] and esophageal carcinoma [9]. Some studies also found that patients with HCC recurrence usually get higher levels of CRP [10,11]. However, to our knowledge, the detailed research regarding the correlation between serum CRP and the recurrence of HCC in patients with chronic HBV infection has not been found yet.

Since effective treatment of recurrence is important in prolonging survival after resection of HCC, we retrospectively collected perioperative variables and preoperative serum CRP levels of patients undergoing curative hepatectomy to explore the clinical correlation between serum CRP and different types of HCC recurrence during the follow-up period. Information from our study would allow the early prediction of the HCC recurrence and may provide clues for understanding the underlying etiological mechanisms of this disease.

## Materials and Methods

### Study population

In the full year of 2008, total 284 patients with chronic HBV infection underwent liver resection for HCC at Shandong Cancer Hospital and Institute. After the exclusion of patients who died in the perioperative period (n = 10), patients who received a noncurative resection (n = 7), patients with incomplete follow up data (n = 14), and patients who underwent preoperative transarterial chemoembolization (TACE) for HCC (n = 21), the remaining 232 cases formed the analysis population in our study.

### Surgical procedures

When performed the primary hepatectomy, all patients demonstrated neither hilar nodal involvement nor extrahepatic metastases. The selection criteria and type of operative procedure were determined according to preoperative indocyanine green retention rate at 15 minutes (ICG R15). Patients with an ICG R15 less than 35% were selected for anatomic resection and ICG-R15 of 35% or more indicated limited resection. Anatomic resection included hemi-hepatectomy, segmentectomy, and subsegmentectomy or more based on Couinaud’s classification.

### Patient follow-up

All patients received routine follow-up for serum alphafetoprotein (AFP) level and ultrasonography monthly for the first year and every 3 months thereafter. The patients also received enhanced computerized tomography (CT) scans at 6-month intervals. Suspected HCC recurrence was confirmed using CT scan, hepatic angiography, and in some cases postlipiodol CT scan or percutaneous biopsy. Additional examinations, such as chest radiograph, chest CT scan, or bone scan, were also performed if there was any sign of extrahepatic recurrence. According to HCC recurrence time, we defined HCC recurrence as early recurrence (ER) and late recurrence (LR) using 24 months as cutoff, which was also adopt in previous studies [12,13].

### Data collection

We retrospectively collected patients’ serum CRP data from original medical records and the follow-up data from our hospital cancer patients’ follow-up database. In current study, blood samples were obtained immediately prior to the date of surgery to exclude other inflammatory effect of preoperative invasive examinations. Serum CRP level was measured using a high sensitivity assay on a Turbidimetrie, Modular-System (Roche Mannheim, Germany). All follow-up data was collected before 31 December 2013. We declared that all data underlying the findings are fully available without restriction. All relevant data were within the paper and its supporting information file (S1 Material).

### Ethics Statement

This was a retrospective study making use of data already collected. All data used in this report were routine clinical data collected in the process of diagnosis and treatment. The processing and analysis of data was done after anonymization. National legislation and the ethical committee of Shandong Cancer Hospital and Institute approved this retrospective study.

### Statistical analysis

**Figure 1:**
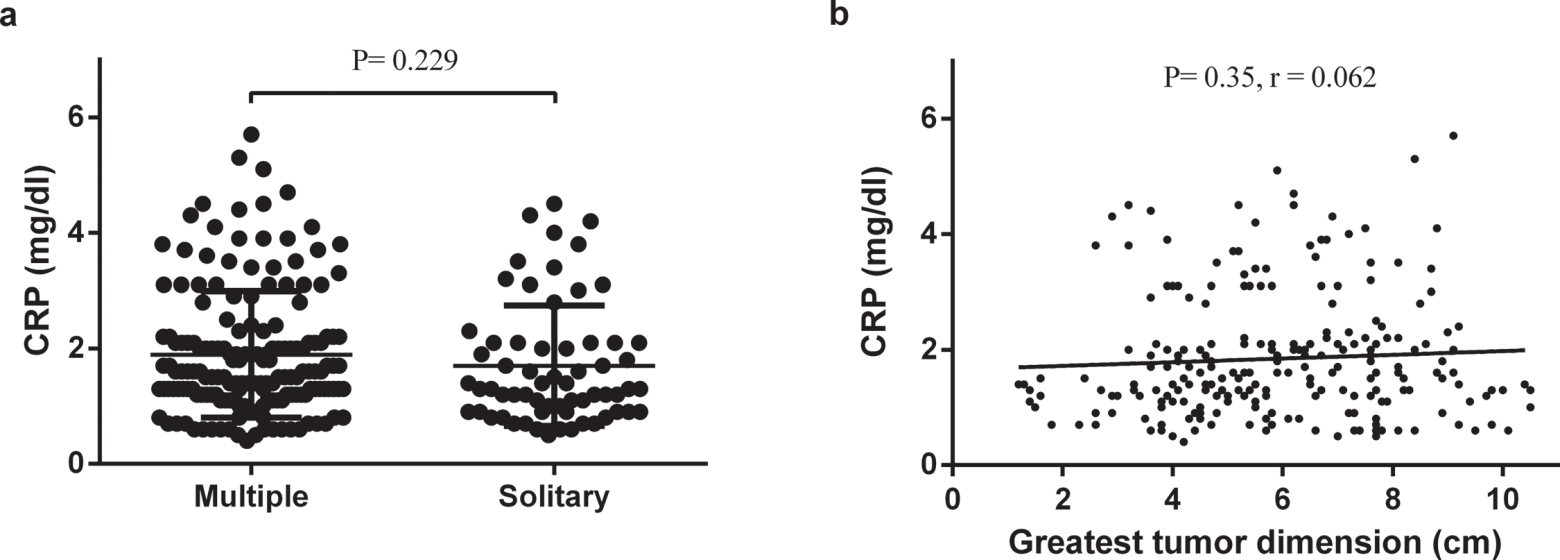
The correlation between CRP and different clinicopathological features groups. (a) Distribution of CRP in both multiple tumors group and solitary tumor group. No significant difference of CRP level was found between the two groups (P = 0.229). (b) The correlation between preoperative serum CRP and the maximal tumor dimensions. This scatter plot showed that preoperative serum CRP levels were not positive correlation with the maximal tumor dimensions (P = 0.35, r = 0.062).

**Figure 2:**
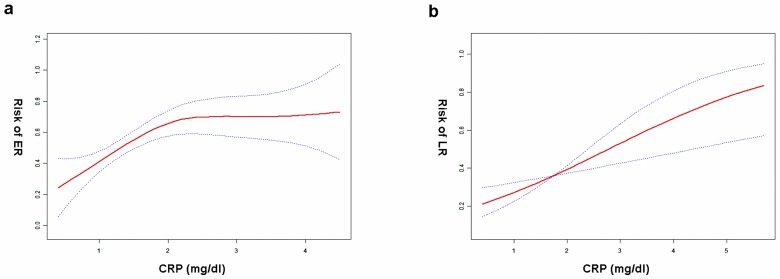
The association between preoperative serum CRP and the risk of HCC recurrence following curative hepatectomy. (a) CRP and ER^a^. A nonlinear relationship between CRP and risk of ER was observed after adjusting for confounding factors. (b) CRP and LR^b^. A linear relationship between CRP and risk of LR was observed after adjusting for confounding factors. ^a^: Adjusted for age, maximal tumor dimension, invasion to the portal vein and histological differentiation. ^b^: Adjusted for age, cirrhosis, maximal tumor dimension and tumor number.

**Figure 3:**
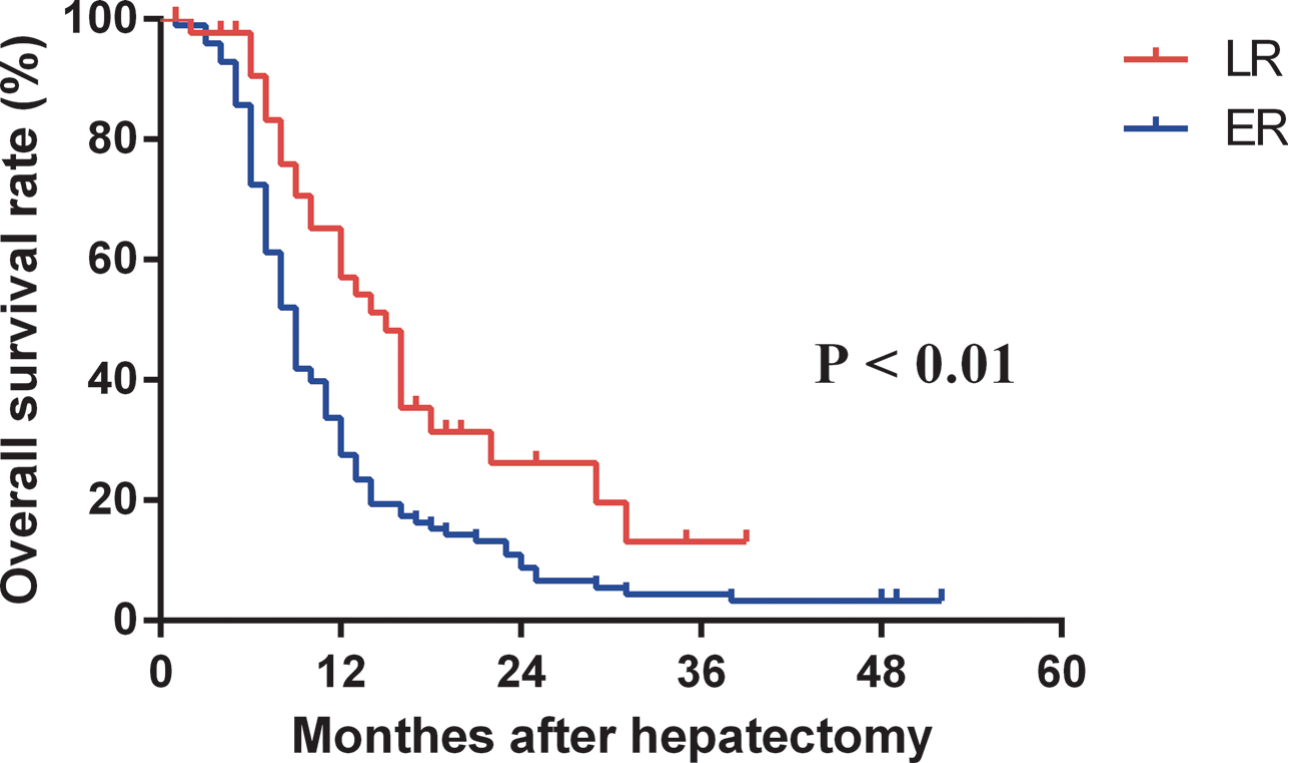
The overall survival curves in patients with ER and LR. Patients with ER had a lower overall survival rates after recurrence than those with LR (P < 0.01).

**Figure 4:**
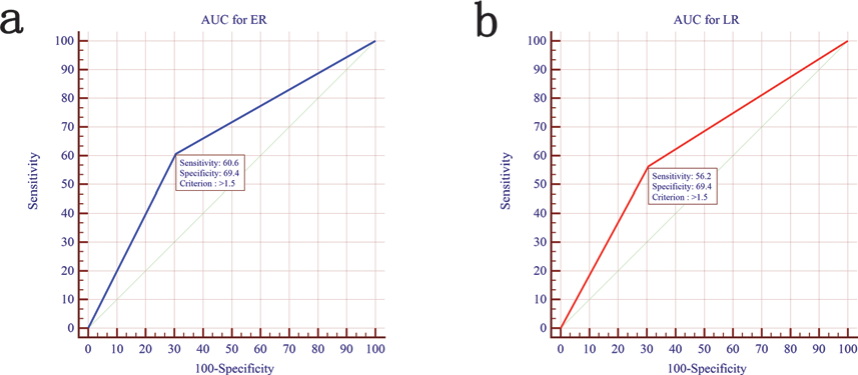
Receiver-operating characteristic (ROC) of the optimal cutoff values for preoperative serum CRP in predicting ER and LR. (a) Preoperative serum CRP as indicator of ER with the area under the curve (AUC) of 0.65 (95% CI, 0.58–0.72) and the optimal cutoff point was 1.5 mg/dl with a sensitivity of 60.6% and a specificity of 69.4%. (b) Preoperative serum CRP as indicator of LR with the AUC of 0.63 (95% CI, 0.54–0.71). The optimal cutoff point was 1.5 mg/dl with a sensitivity of 56.2% and a specificity of 69.4%.

**Figure 5:**
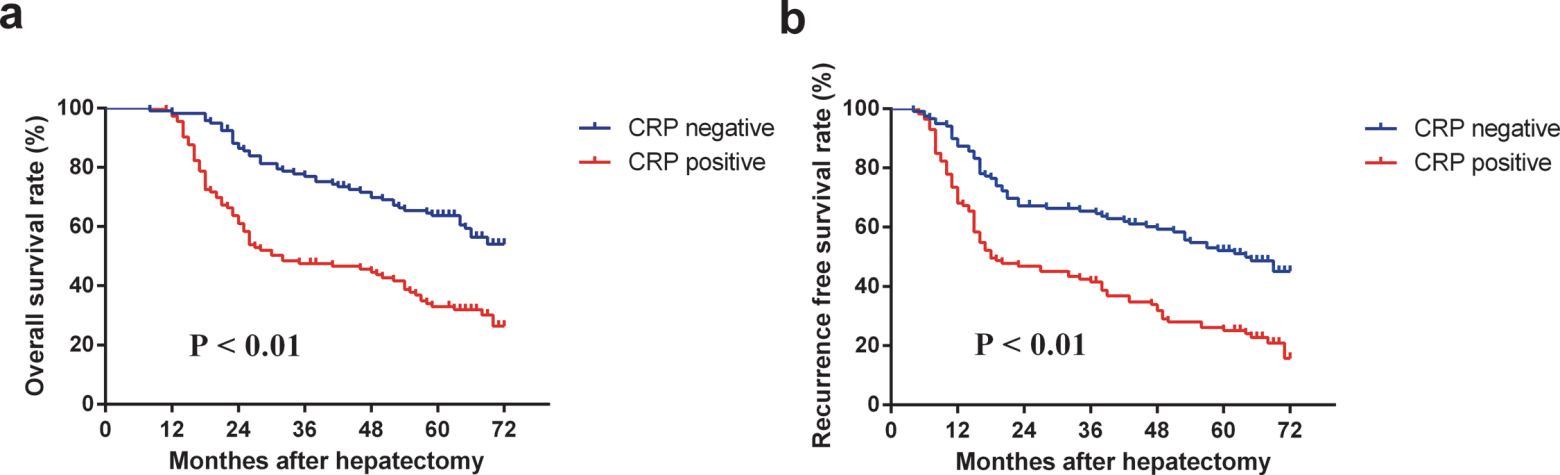
The recurrence-free survival curves and the overall survival curves in patients with serum CRP negative and positive. (a). The recurrence-free survival rates in the CRP negative group were significantly higher compared with those in the CRP positive group (P < 0.01). (b) The overall survival rates in the CRP negative group were significantly higher compared with those in the CRP positive group (P < 0.01).

Student’s t test, Pearson Chi-square test and the Fisher’s exact test were used to compare groups (Table 1). The Student’s t test was used to compare quantitative variables in different groups (Fig. 1A), the association between serum CRP and greatest tumor dimension was analyzed by the Pearson’s correlation test (Fig. 1B). The relationship between serum CRP and the risk of ER, LR were explored by the smoothing plot (Fig. 2). We applied a two-piecewise linear regression model to examine the saturation effect of the serum CRP on risk of ER according to the smoothing plot (Table 2). An inflection of CRP, at which the relationship between CRP and the risk of ER began to change and become not eminent, was determined using a trial method. The latter was to move the trial inflection point along a pre-defined interval and detect the inflection point that gave the maximum model likelihood. Comparisons between the CRP negative and CRP positive groups were performed with the student’s t test and the chi-square test (Table 3). The overall survival (OS) were calculated using the Kaplan-Meier method, and the difference between the groups were assessed by the log-rank test (Fig. 3). Receiver operating characteristics (ROC) curves were used to define optimal cutoff value, sensitivity and specificity (Fig. 4). The cumulative recurrence-free survival (RFS) and OS were also calculated using the Kaplan-Meier method and compared by the log-rank test (Fig. 5). The exploratory subgroup analyses were performed and P for interaction were from log likelihood ratio test comparing two nested models (Table 4).

All data were double entered and then exported to tab-delimited text files. Probability values of less than 0.05 were considered statistically significant. Statistical analyses were performed with R (http://www.R-project.org).

**Table 1:** Clinicopathological features of 232 patients who received curative hepatectomy.

**Variable**	**No recurrence**	**ER**	**LR**	**P^1^ value**	**P^2^ value**
N (%)	85	99	48		
CRP (mg/dl)	1.5 ± 0.9	2.0 ± 1.0	2.2 ± 1.4	<0.001*	<0.001*
Age (yrs)^a^	50.1 ± 6.6	51.4 ± 6.7	50.8 ± 7.1	0.184	0.562
Gender (male: female)	73:12	80:19	41:7	0.359	0.941
Child’s class (A: B)	58:27	76:23	34:14	0.195	0.755
Cirrhosis (present: absent)	75:10	91:8	45:3	0.402	0.304
ALT (IU/L)^a^	77.6 ± 30.0	76.7 ± 27.0	71.9 ± 30.7	0.834	0.301
AST (IU/L)^a^	65.5 ± 26.3	63.3 ± 25.9	62.7 ± 26.5	0.555	0.554
Total bilirubin (mg/dL)^a^	1.0 ± 0.3	1.0 ± 0.3	1.0 ± 0.3	0.327	0.321
Albumin (g/dL)^a^	3.9 ± 0.2	3.9 ± 0.2	4.0 ± 0.2	0.855	0.207
Platelet count (10^4^/mm^3^)^a^	14.5 ± 4.0	15.4 ± 4.2	15.4 ± 3.9	0.139	0.241
Maximal tumor dimension (cm)^a^	5.4 ± 2.0	6.2 ± 2.0	6.2 ± 2.3	0.008*	0.030*
Tumor number (multiple: solitary)	56:29	72:27	41:7	0.314	0.015*
Micrometastasis (present: absent)	44:41	51:48	22:26	0.973	0.511
Macroscopic vascular invasion (present: absent)	40:45	44:55	22:26	0.723	0.892
Invasion to the portal vein (present: absent)	12:73	28:71	9:39	0.020*	0.471
Anatomic resection (yes: no)	56:29	61:38	34:14	0.549	0.558
Histology, n (%)				0.025*	0.784
Well differentiated	32 (37.6)	25 (25.3)	16 (33.3)		
Moderately differentiated	32 (37.6)	31 (31.3)	21 (43.8)		
Poorly differentiated	21 (24.7)	43 (43.4)	11 (22.9)		

ER: early recurrence; LR: late recurrence; CRP: C-reactive protein; ALT: alanine aminotransferase; AST: aspartate aminotransferase.

^a^ Values shown are the mean±standard deviation

* Significant difference.

P^1^ value: comparison between ER group and control group, P^2^ value: comparison between LR group and control group.

**Table 2:** Threshold effect analysis of CRP on different types of HCC recurrence following curative hepatectomy using piece-wise linear regression.

	**Crude OR (95% CI)**	**p-value**	* **Adjusted OR (95% CI)**	**p-value**
ER				
CRP≦2.1(mg/dl)	3.5 (1.7, 7.5)	0.001	3.5 (1.6, 7.6)	0.001
CRP>2.1(mg/dl)	0.8 (0.3, 2.1)	0.614	0.8 (0.2, 2.7)	0.703
LR	1.7 (1.2, 2.4)	0.001	1.8 (1.2, 2.5)	0.002

Crude: no adjustment.

* Adjusted: In ER group, characters of age, maximal tumor dimension, invasion to the portal vein and histological differentiation were adjusted. In LR group, characters of age, the maximal tumor dimension and tumor number were adjusted.

**Table 3:** Patient characteristics and demographics between CRP negative and CRP positive groups.

**Variable**	**CRP negative**	**CRP positive**	**P value**
N (%)	119 (51.3)	113 (48.7)	
Age (yrs)^a^	50.7 ± 7.0	50.9 ± 6.5	0.842
Gender (male: female)	100:19	94:19	0.862
Child’s class (A: B)	83:36	85:28	0.351
Cirrhosis (present: absent)	86:33	82:31	0.960
ALT (IU/L)^a^	78.1 ± 28.8	73.9 ± 29.0	0.261
AST (IU/L)^a^	62.9 ± 25.7	65.1 ± 26.5	0.517
Total bilirubin (mg/dL)^a^	1.0 ± 0.3	1.0 ± 0.3	0.868
Albumin (g/dL)^a^	4.0 ± 0.2	3.9 ± 0.2	0.831
Platelet count (10^4^/mm^3^)^a^	14.7 ± 3.9	15.5 ± 4.3	0.178
Maximal tumor dimension (cm)^a^	5.6 ± 2.4	6.1 ± 1.7	0.068
Tumor number (multiple: solitary)	82:37	87:26	0.166
Micrometastasis (present: absent)	65:54	52:61	0.190
Macroscopic vascular invasion (present: absent)	55:64	51:62	0.868
Invasion to the portal vein (present: absent)	23:96	26:87	0.492
Anatomic resection (yes: no)	83:36	85:28	0.126
Histology, n (%)			0.295
Well differentiated	38 (31.9)	35 (31.0)	
Moderately differentiated	45 (37.8)	39 (34.5)	
Poorly differentiated	36 (30.3)	39 (34.5)	

CRP: C-reactive protein; ALT: alanine aminotransferase; AST: aspartate aminotransferase.

^a^ Values shown are the mean±standard deviation.

**Table 4:** Effect of different clinicopathological features on association between CRP and HCC recurrence in exploratory subgroups.

	**ER**						**LR**		
		
		**CRP< 2.1**			**CRP≥ 2.1**				
	**N**	**OR(95%CI)**	**P**	**N**	**OR(95%CI)**	**P**	**N**	**OR(95%CI)**	**P**
Maximal tumor dimension			<0.001*			0.002*			0.766
≦5cm	21	−0.1 (−0.4, 0.1)		4	0.3 (0.0, 0.5)		16	0.2 (0.0, 0.4)	
>5cm	42	0.6 (0.4, 0.8)		32	−0.2 (−0.4, 0.0)		32	0.3 (0.1, 0.4)	
Tumor number			0.112			0.448			0.013^*^
Multiple	17	0.0 (−0.4, 0.4)		10	−0.1 (−0.4, 0.1)		7	0.3 (0.2, 0.5)	
Solitary	46	0.4 (0.1, 0.6)		26	0.0 (−0.2, 0.2)		41	−0.1 (−0.4, 0.2)	
Invasion to the portal vein			0.498			0.797			0.656
Present	17	0.4 (−0.1, 0.9)		11	0.0 (−0.3, 0.3)		9	0.2 (−0.2, 0.5)	
Absent	46	0.2 (0.0, 0.4)		25	−0.1 (−0.3, 0.1)		39	0.3 (0.1, 0.4)	
Histology			0.625			0.837			0.369
Well differentiated	16	0.3 (0.0, 0.6)		8	0.0 (−0.3, 0.3)		17	0.1 (−0.2, 0.4)	
Moderately differentiated	29	0.1 (−0.2, 0.4)		22	−0.1 (−0.4, 0.2)		18	0.3 (0.1, 0.5)	
Poorly differentiated	18	0.3 (0.0, 0.7)		6	−0.1 (−0.4, 0.2)		13	0.2 (−0.1, 0.5)	

* Significant difference, association between CRP and HCC recurrence was significant different in subgroup analyzes

## Results

Clinicopathologic characteristics of 232 patients who received curative hepatectomy are summarized in Table 1. Until the endpoint of the study, 85 of the 232 patients had no evidence of HCC recurrence with a median follow up of 65 months (ranging from 21 to 72 months) and 147 patients were documented as having HCC recurrence with a median follow up of 16 months (ranging from 4 to 71 months). Of these patients with HCC recurrence, 99 patients (67.3%) presented ER and 48 patients (32.7%) presented LR. Serum CRP in either ER or LR group was significantly higher than that in no recurrence group. A larger maximal tumor dimension, the presence of portal vein invasion, and the poorly differentiated histology were significantly correlated with ER, whereas a larger maximal tumor dimension and multiple tumors were significantly associated with LR. Patients in ER group or LR group did not differ significantly from the 85 patients without HCC recurrence in relation to age, gender, Child-Pugh grade, ALT, AST and total bilirubin.

Suspicious factors associated with serum CRP levels were also analyzed. The mean serum CRP levels in patients with multiple tumors was 1.7mg/dl, this was not significantly different with that in patients with solitary tumor (1.9md/dl, P = 0.229, Fig. 1A). In addition, there was no significant correlation between serum CRP level and the maximal tumor dimension (P = 0.35, r = 0.062, Fig. 1B).

Multivariate regression analyses were performed to estimate the independent relationship between serum CRP levels and ER or LR, with adjustment for potential confounders (Table 2). After adjusting for age, maximal tumor dimension, the presence of portal vein invasion, and the outcome of histology, a nonlinear relationship between the serum CRP levels and the risk of ER was observed (Fig. 2A). The risk of ER increased with serum CRP level up to the turning point (2.1mg/dl) (OR = 3.5, 95% CI 1.6–7.6, P = 0.001). When cryoprecipitate level was ➮2.1mg/dl, nor was the level of serum CRP associated with the risk of ER (OR = 0.8, 95% CI 0.2–2.7, P = 0.703). Somewhat differently, serum CRP levels showed a linear correlation of the incidence of LR, after adjusting for age, maximal tumor dimension and tumor numbers (Fig. 2B). The risk of LR increased as serum CRP level raised (OR = 1.8, 95% CI, 1.2–2.5, P = 0.002).

Patients with ER also had a worse overall survival than those with LR. The overall 1-year, 3-year and 5-year survival rates after early recurrence were 54.2%, 26.2%, and 16.6%, respectively (median survival, 16 months), which were worse than the corresponding survival rates of 88.2%, 43.2%, and 27.0% (median survival, 30 months) after late recurrence (Fig. 3, P < 0.01).

Receiver operating characteristic curve analysis was used to verify the prediction ability of the serum CRP level for different types of HCC recurrence. The AUC (area under the curve) for serum CRP level in predicting ER and LR were 0.65 (95% CI, 0.58–0.72) (Fig. 4A) and 0.63 (95% CI, 0.54–0.71) (Fig. 4B), respectively. The optimal cutoff point for serum CRP level was 1.5 mg/dl in predicting ER, with a sensitivity of 60.6% and a specificity of 69.4%, and the optimal cutoff point for serum CRP level was also 1.5 mg/dl in predicting LR, with a sensitivity of 56.2% and a specificity of 69.4%.

According to this optimal cutoff value, patients with preoperative serum CRP levels ≤1.5 mg/dl were assigned to the CRP negative group, whereas patients with preoperative serum CRP levels >1.5 mg/dl were assigned to the CRP positive group. By this definition, CRP negative was recognized in 119 patients (51.3%). Conversely, CRP positive was recognized in 113 patients (48.7%). Among variables that might affect patient survival or HCC recurrence, there was no statistical significant difference in the basic characteristics between the two groups (Table 3).

The recurrence-free survival rate in the CRP negative group was significantly higher compared with that in the CRP positive group (Fig. 5A, P < 0.01). The 1-year, 3-year, and 5-year cumulative recurrence-free survival rates were 94.0%, 76.0%, and 65.7% respectively in the CRP negative group and 73.6%, 47.7%, and 31.5% respectively in the CRP positive group. Similarly, the CRP negative group also had a higher overall survival rate compared with the CRP positive group (Fig. 5B, P < 0.01). The 1-year, 3-year, and 5-year overall survival rates were 100%, 89.8%, and 77.3% respectively in the CRP negative group and 97.3%, 55.1%, and 40.9% respectively in the CRP positive group.

Additional exploratory subgroup analyses were conducted to evaluate the consistency of the results (Table 4). In ER group, there was evidence for an interaction between serum CRP and the maximal tumor dimension. As compared to those with maximal tumor dimension≦ 5cm, odds ratio for CRP was significant from those with maximal tumor dimension> 5cm (P < 0.01 for interaction). In LR group, the test for interaction was significant for tumor numbers (P = 0.013 for interaction). Odds ratio for CRP was significant higher in multiple tumors patients. With respect to other exploratory factors, there was no evidence that either HCC invasion to the portal vein or histology differentiation status significantly modified the odds ratio for CRP in both ER and LR groups.

## Discussion

In the current study, after investigating detailed preoperative serum CRP levels contributing to ER and LR respectively, a linear relationship existed between preoperative serum CRP and LR, while this inflammation-related cytokine had a nonlinear relationship with ER. In addition, we got the conclusion that lower preoperative serum CRP level was significantly correlated with recurrence-free survival and overall survival in patients with chronic HBV infection. However, the optimal cutoff value of preoperative serum CRP for both ER and LR was 1.5 mg/dl instead of 1.0 mg/dl, which was always adopted in previous studies [14,15].

As reported, CRP can promote tumor progression through a variety of possible ways, including anti-apoptotic activity and tumorigenic potency, T-cell function damage, increased levels of serum angiogenic factors, and resistance to chemotherapy[9,16]. On the other hand, CRP is also upregulated by cytokines such as IL-6 and tumor necrosis factor, and this upregulation is considered to be a systemic reaction to tumor progression[17,18]. Actually, issues of whether aggressive tumor behavior prompts a prognostically detrimental inflammatory reaction or whether inflammation per se drives tumor progression remains to be further studied[19,20]. Notably, it is beneficial to elucidate or investigate the complex relation between CRP and HCC recurrence by using detailed preoperative serum CRP levels, instead of cutoff value.

It has been found recently that there may be differences in the biologic origin and behavior between ER and LR[21]. In order to further verify this hypothesis, we investigated detailed preoperative serum CRP levels contributing to ER and LR, separately. Of interest, a saturation effect for preoperative serum CRP of 2.1 mg/dl existed for ER, whereas a linear relationship existed between preoperative serum CRP and LR. To the best of our knowledge, few population-based clinical studies to date have provided evidence of a quantitative regression relation between preoperative serum CRP and HCC recurrence in patients with chronic HBV infection. In addition, we also found that patients with ER had a lower overall survival than those with LR. Different prognosis of ER and LR further supported the hypothesis mentioned above. These results provided some insights for further studying the influence of inflammation cytokines on HCC recurrence in patients with chronic HBV infection. Previous studies reported that preoperative serum CRP over 1.0 mg/dl could significantly increase the risk of HCC recurrence, in which most research subjects were HCV patients [14,15]. Different from these studies, the current study demonstrated that the optimal cutoff point of preoperative serum CRP to predict both ER and LR was 1.5 mg/dl, in patients with chronic HBV infection. Although CRP concentration exceeding 1.0 mg/dl was considered to indicate the presence of a systemic inflammatory response based on findings from previous investigation[22], it is noteworthy that this cutoff value may not be applicable to the prediction of HCC recurrence in patients with chronic HBV infection. Compared to previous studies, a higher optimal cutoff value of serum CRP in predicting HCC recurrence in our study may be explained by the following two reasons. First, elevated serum CRP levels can be affected by coexistent cirrhosis[23,24], which is common in patients with chronic HBV infection. Second, chronic HBV infection can activate the innate immunity and promotes various inflammatory reactions and triggers the release of inflammatory cytokines as well as CRP[25].

In contrast to the close correlation between serum CRP level and unfavorable tumor factors, as shown in previous study[15], the elevated serum CRP level itself is not directly linked with these unfavorable tumor factors, such as maximal tumor size and tumor numbers, in the current analysis. Sieghart et al.[26] also showed that elevated serum level of CRP is strongly associated with dismal prognosis, but it also independently from tumor characteristics. In fact, whether serum CRP level is associated with tumor characteristics remains controversial. Because of the complex association between HCC and serum CRP level, it is reasonable to accept that different conclusions from different study populations.

Our study demonstrated that preoperative serum CRP positive (>1.5 mg/dl) led to a poorer overall survival and recurrence-free survival in patients with chronic HBV infection. The mechanistic role of tumor-related CRP in HCC recurrence is largely unclear. However, significant correlation between higher preoperative serum CRP level and poorer outcome has been validated in many clinical studies[14,15,27]. On the basis of above clinical findings, some researchers hypothesized that high serum CRP levels may reflect the aggressiveness of the tumor or cytologic tumor spread[14,15]. These hypotheses also could reasonably explain the association between high serum CRP level and high recurrence rate and low survival rate, found in the current study.

Interesting enough, subgroup analyses suggested the prognostic relevance of serum CRP was affected by maximal tumor size and tumor numbers. For HCC patients with elevated serum CRP, the risk of ER rise significantly when the maximal tumor dimension was more than 5cm. Besides, elevated serum CRP together with multiple tumors increase the risk of LR significantly. These findings strongly support the hypothesis that complex internal relationship exists between tumor progression and CRP, as mentioned above. From another point of view, the association between CRP and tumor characteristics in ER patients varied from that in LR patients reflected some diversities of these two types of HCC recurrence. In clinical practice, it is suggested that elevated serum CRP levels combined with tumor characteristics can predict different types of HCC recurrence more effectively, though the exact underlying mechanism remains to be further investigated.

In conclusion, preoperative serum CRP has a potential as an effective biomarker in the early prediction of HCC recurrence following curative hepatectomy in patients with chronic HBV infection. However, the association between this cytokine and HCC recurrence is more complicated than ever expected, and further studies are needed to examine the exact biological role of CRP on different types of HCC recurrence. The causal role of CRP in HCC progression merits further investigations in exploring potential applications for HCC recurrence prevention.

## Supporting Information

S1 Material Data regarding patients’ characters. (XLSX)
